# Metabolome progression during early gut microbial colonization of gnotobiotic mice

**DOI:** 10.1038/srep11589

**Published:** 2015-06-29

**Authors:** Angela Marcobal, Tahir Yusufaly, Steven Higginbottom, Michael Snyder, Justin L. Sonnenburg, George I. Mias

**Affiliations:** 1 Department of Microbiology and Immunology, Stanford University School of Medicine, Stanford, California, USA; 2 Department of Biochemistry and Molecular Biology, Michigan State University, East Lansing, Michigan, USA; 3 Department of Genetics, Stanford University School of Medicine, Stanford, CA, USA

## Abstract

The microbiome has been implicated directly in host health, especially host metabolic processes and development of immune responses. These are particularly important in infants where the gut first begins being colonized, and such processes may be modeled in mice. In this investigation we follow longitudinally the urine metabolome of ex-germ-free mice, which are colonized with two bacterial species, *Bacteroides thetaiotaomicron* and *Bifidobacterium longum*. High-throughput mass spectrometry profiling of urine samples revealed dynamic changes in the metabolome makeup, associated with the gut bacterial colonization, enabled by our adaptation of non-linear time-series analysis to urine metabolomics data. Results demonstrate both gradual and punctuated changes in metabolite production and that early colonization events profoundly impact the nature of small molecules circulating in the host. The identified small molecules are implicated in amino acid and carbohydrate metabolic processes, and offer insights into the dynamic changes occurring during the colonization process, using high-throughput longitudinal methodology.

The human body is populated by a dense array of microorganisms that constitute a dynamic source of macromolecules and small molecules (metabolites)^1^. These bacterial-origin metabolites are absorbed continuously by the host and integrated into their systemic circulation. Efforts are in progress to catalogue specific microbial populations in healthy and pathological human states, including the extensive research from the Human Microbiome Project^2^,^3^,^4^,^5^,^6^, and new directions in integrating such information for personalized medicine approaches^7^,^8^,^9^. The microbiome has been shown to have multiple connections to human physiology, including the effects of energy modulation and connections to obesity, metabolic disorders and drug efficacy^10^. The effect of intestinal dysbiosis has been associated with various diseases, including obesity, diabetes, Crohn’s and celiac diseases^11^,^12^,^13^,^14^,^15^,^16^,^17^,^18^. The influence of diet on the constitution of the microbiome has been also explored, both in the context of mechanism discovery, as well as effecting physiological changes, such as treatment of disease, obesity reduction as well as modulating drug efficacy and toxicity^10^,^19^,^20^. The study of the microbiome and the implementation of pharmacomicrobiomics^20^ lends itself to applications relevant to the recent efforts on precision medicine announced by the National Institutes of Health^21^. The interplay between host-microbiome interactions is being extensively modeled, at all scales, starting from individual biochemical interactions and protein/gene associations to systems-level implementations that predict global effects of host-microbiome modulation, colonization and diet, including genome-scale metabolic models (GEMs)^22^.

Additional investigations have studied the processes that drive the development of adult-like bacterial composition in the gut, beginning from a “sterile” environment at birth^23^,^24^. Studies involving 16 S ribosomal RNA (rRNA) based sequencing^25^ and metatranscriptomic or proteomic analyses^26^,^27^,^28^ have reported the involvement of conditions at birth and infant diet in the complex development of gut microbiota observed during the first months of life^29^. The 16 S rRNA-based studies revealed that in the intestinal microbiota of healthy adult humans the majority (>99%) of detected phylotypes belonged to two bacterial divisions: *Bacteroides* and *Firmicutes*^30^. These relative proportions of these bacterial populations have already been implicated in studies contrasting children’s diets^31^.

The specific mechanisms that select for particular groups of bacteria in the infant gut remain largely unknown. To mimic the development of the bacterial flora in the sterile gut of a newborn, simplified mice models, like germ free (GF) mice, have been employed^32^ and Gnotobiotic mice (ex-GF mice) colonized with specific sets of microorganisms or full microbial communities have been shown to be useful models in the study of symbiont–host interactions. The dynamics of microbial colonization events early in life upon the host metabolome are also still under investigation. Metabolome changes in feces from germ free mice during the process of acclimatization to non-germ free environment have been previously evaluated^33^. Martin *et al*. studied fecal metabolic differences between conventional mice and germ free mice colonized with a mix of seven microbes isolated from baby fecal samples^34^. Their investigations revealed differences in metabolite content and proportion at individual time points post colonization. We further extend such approaches to the use of time-series correlation trends to follow the effects of bacterial acquisition as described below.

To extract more information about the colonization process new systems biology methodologies are now being implemented, aided by recent advent of metabolomics technologies (e.g. Nuclear Magnetic Resonance (NMR), gas chromatography/mass spectrometry (GC-MS), or ultra-performance liquid chromatography/mass spectrometry (UPLC/MS)^35^,^36^), which provide a new toolkit, enabling the profiling and monitoring of all the metabolite components in a given sample. These high-throughput approaches capture the intensities of thousands of components and reveal that host metabolomes are directly impacted by the presence of gut bacteria^25^. The identity and function of microbes colonizing the gut has a direct impact on the small molecules, metabolites, that are produced^34^,^37^. Many of these metabolites, which are still structurally uncharacterized, are taken up into host circulation where they can interact with tissues throughout the body, be co-metabolized by enzymes including those in the liver, and ultimately are excreted through the kidney into urine. Comparison of urine metabolic profiles from conventional and germ free rats and mice^38^,^39^ revealed an important contribution of the gut microbiota in liver or kidney metabolism, and a connection to adaptive immune responses^40^. Other studies have verified how the presence of bacteria impact the colonic luminal metabolome^41^, as well as some endogenous metabolite levels^42^.

In this investigation we address the direct response in a germ free environment to bacterial colonization, and evaluate changes in host urine metabolome over time. To mimic the interaction of bacteria-sterile gut we use a gnotobiotic mice model, colonizing germ free mice with *Bacteroides thetaiotaomicron* (*Bt*) and *Bifidobacterium longum* (*Bif. longum*), commonly present in the infant gut^32^,^43^,^44^. The urine metabolome was profiled over multiple time points (spanning 25 days), once in germ free (GF) used as a reference and every five days in colonized mice, using UPLC-MS technologies. One of the major hurdles in analyzing such time-course data is accounting for the sampling. Missing intensity information for mass features in mass spectrometry spectra leads to unevenness in the sampling, even if the experiment was designed to sample at regular intervals. To allow for this, we applied a method that was initially developed for temporal data in astronomy^45^,^46^,^47^, and later extended to other time series, including biological and omics longitudinal data^7^,^48^,^49^,^50^,^51^,^52^,^53^. The approach takes the time variable into account, using classification of each signal by autocorrelation and then pattern matching to reveal the underlying collective behavior and is generalizable to longer time series and other omics (Yusufaly and Mias in preparation). The complex data spectra were analyzed in order to identify metabolites and their temporal trends, to identify and classify the various patterns corresponding to the process of bacterial colonization. Interesting features that show the response to microbiota introduction in the host metabolome include both continuous trends and sudden increases and decreases in metabolite intensity levels with a range of metabolic pathways observed (related to immune responses, amino acid and carbohydrate processing pathways).

## Results

**Figure 1:**
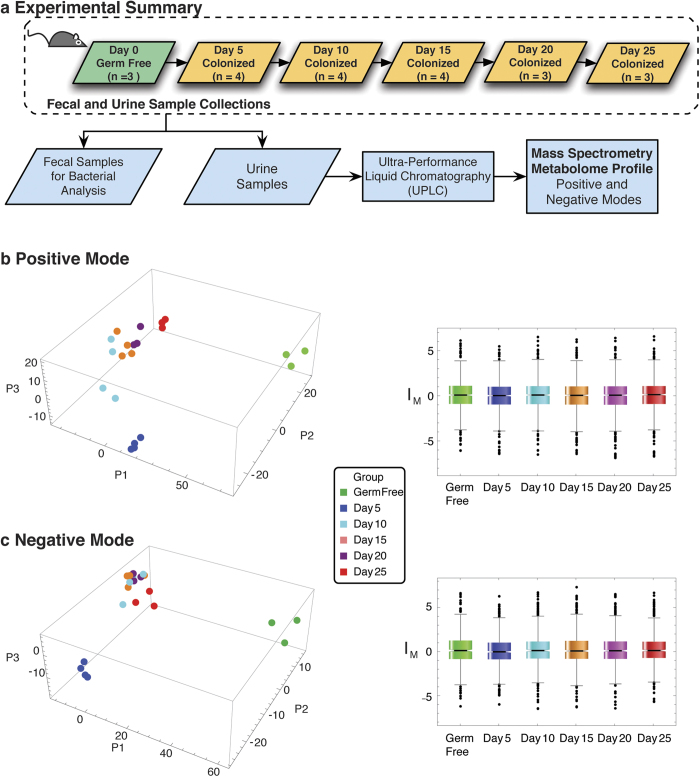
Inoculation of Germ Free Mice. **(a)** Germ free mice were inoculated on Day 0 with *Bacteriodes. thetaiotaomicron* and *Bifidobacterium longum* by oral gavage ingestion. Urine and fecal samples were processed for metabolome and colony profiling respectively from these mice on days 5, 10, 15, 20, 25. Principal Component Analysis in both Positive (**b**) and Negative (**c)** acquisition mode full metabolome results reveal that the mice samples aggregate into groups, with the Germ Free and Day 5 groups well separated. The components account for 81% and 80% of the variances in Positive and Negative modes respectively. The corresponding normalized distributions are shown on the right panels displaying similar profiles, where intensities I_M_ have been standardized (with median and median absolute deviation), indicating symmetry in approach in positive and negative modes. See also Supplementary Fig. S1, Supplementary Table S1.

To evaluate changes due to bacterial colonization in germ free mice, the study followed the global changes in metabolomics from urine samples in Swiss-Webster germ free (GF) mice, after colonization. The urine samples were essentially used as an output signature of changes caused by the bacterial colonization. The small molecules in mouse urine were profiled using mass spectrometry over the course of 25 days (Fig. 1a) at five days intervals.

The mice, initially all GF, were placed inside gnotobiotic isolators. One group of GF mice (n = 3) was used as a reference for comparison. The other group of initially GF mice (n = 4) was colonized on Day 0 using oral gavage with 10^8^ cfu of *Bt* (VPI-5482) and 10^8^ cfu of *Bif. longum* (NCC2705). Urine samples (used for metabolome profile) and feces (used for verifying the colonization composition) from GF mice were collected on Day 0, and similarly from the bi-associated mice on Days 5, 10, 15, 20 and 25 after their colonization. The bacterial composition in the bi-associated mice was determined by plating assays of fecal samples, and did not fluctuate after Day 5 (Supplementary Fig. S1), showing a relatively higher proportion of *Bt* (75–95% range). The urine samples collected were processed using high affinity liquid chromatography and mass spectrometry (LC-MS), using an Exactive (Thermo Fisher) mass spectrometer, with the electrospray operated in both positive and negative ion modes. The mass spectra were aligned in both retention times and masses (see Methods), resulting in the overall detection of 3245 mass features of interest (1555 and 1692 detected in negative and positive mode respectively).

The processed mass spectra were then analyzed to obtain underlying trends (as outlined in Supplementary Fig. S2). Namely, datasets were: (1) analyzed using Principal Components Analysis (PCA) to assess variation between the GF mice and the bi-associated mice on different days, (2) spectrally analyzed to obtain longitudinal patterns and classified into significance categories based on their profile over time, and (3) potential biological significance assessed through the assignment of putative mass identities and pathway analysis (see Methods). In particular:

(1) The PCA of the aligned comprehensive data revealed a clear separation of the mice into three major sets: (i) The GF mice group and (ii) the Day 5 group, which are distinctly separated from (iii) the remaining groups (Days 10–25) which are rather intermixed as one set (Fig. 1b). The corresponding normalized distributions of metabolites of GF mice and each of the bi-associated mice timepoints remained similar across all measurements, both in negative and positive modes, indicative of the robustness of the normalization procedure. The PCA analysis results can account for most of the variability between the mice groups. In particular the total variance accounted by the three components shown in Fig. 1b was ~80% for the positive mode data (variance from [P1,P2,P3]≈[55%,19%,6%]) and similarly ~81% for negative mode data (variance from [P1,P2,P3]≈[59%,18%,4%]).

(2) The normalized data were used to construct time series signals. For each detected mass feature, a time series was constructed using the GF mice dataset as a stable point reference^39^ - an effective “day zero” data point. For a given mass feature, the data from the time points measured in the bi-associated mice (Day 5-Day 20 after inoculation) were all compared to the same corresponding GF entry. Each resulting signal of relative metabolite changes was classified and assigned to one of three classes, if it displayed one of the following significant temporal trends: (I) autocorrelated at lag one (at p < 0.05; bootstrap distribution; n = 100,000 with replacement) where the signal displays correlated behavior (essentially linear) between each sequential time point in the signal; or as spike trends showing maxima (II) or minima (III), i.e. aberrantly high or low levels respectively, as compared to the signal baseline with random fluctuations, (at p < 0.05; based on n = 100,000 bootstrap simulation with replacement).

**Figure 2:**
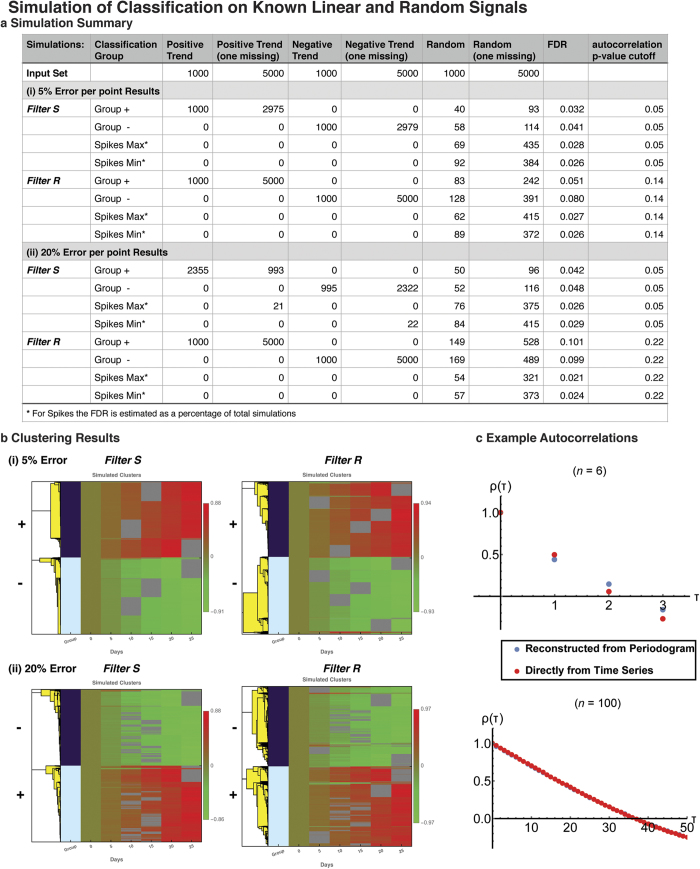
Simulation of Random Time Series. **(a)** Simulations of the autocorrelation classification methodology were performed to assess robustness and reconstruction of temporal trends in known linear signals. The example illustrates a random realization that corresponds to a simulation of: 12,000 linear signals, of equal numbers of positive (+) or negative slope/trend (−), combined with a randomly generated equivalent set of 6000 random signals, and in randomized order. The numbers in each set were equally distributed between having zero or one time point missing (excluding the first one which is used as a reference). Additionally a simulation of 100,000 random signals was used as a background of bootstrap simulation. In particular, for simulations of linear signals with 5% random error per timepoint, *Filter S* corresponds to a strict p < 0.05 cutoff for autocorrelation at lag 1 (random series bootstrap, n = 100,000). In *Filter R* the p-value is relaxed until the entire set of linear trends is recovered. As seen, this results in modest false discovery rates. The simulation was repeated for linear signals with 20% error per timepoint for both filters. The corresponding heatmaps and clustering are shown in **(b)**. **(c)** The reconstruction of autocorrelations from the periodogram, and the exact autocorrelations are shown for a straight signal, for a series of n = 6 and n = 100 time points. For n = 100 the reconstruction is indistinguishable from the exact calculation in the figure.

The above outlined classification approach (see Methods) was also tested in simulations, and found to perform well for six time-points (directly applicable to this project, Fig. 2). The simulations assessed robustness and reconstruction of temporal trends in known linear signals. The linear signals were perturbed through the addition of random noise, either 5% or 10% and also combined with random signals. Each signal set was allowed to have up to one time point missing (except the first time point in all series, which was used as a reference point, in analogy of using the GF mice as a comparison reference point). In the simulations, two filters were used to explore the efficiency of the algorithm, (i) a strict p < 0.05 cutoff for autocorrelation at lag one (*Filter S*) and (ii) a relaxed filter (*Filter R*), in which the p-value was relaxed until the entire set of linear trends was recovered by the classification. The simulation results suggested modest false discovery rates (FDR). In a typical example shown in Fig. 2, for the case of 5% error in the signals, *Filter S* had FDR 0.026–0.041 and *Filter R* recovered all linear signals at p < 0.14 and FDR 0.026–0.08. For 20% errors in the signals, *Filter S* had FDR 0.026–0.048 and *Filter R* recovered all linear signals at p < 0.22 and FDR 0.021–0.101. The heatmaps and clustering corresponding to the simulation results are shown in Fig. 2b.

**Figure 3:**
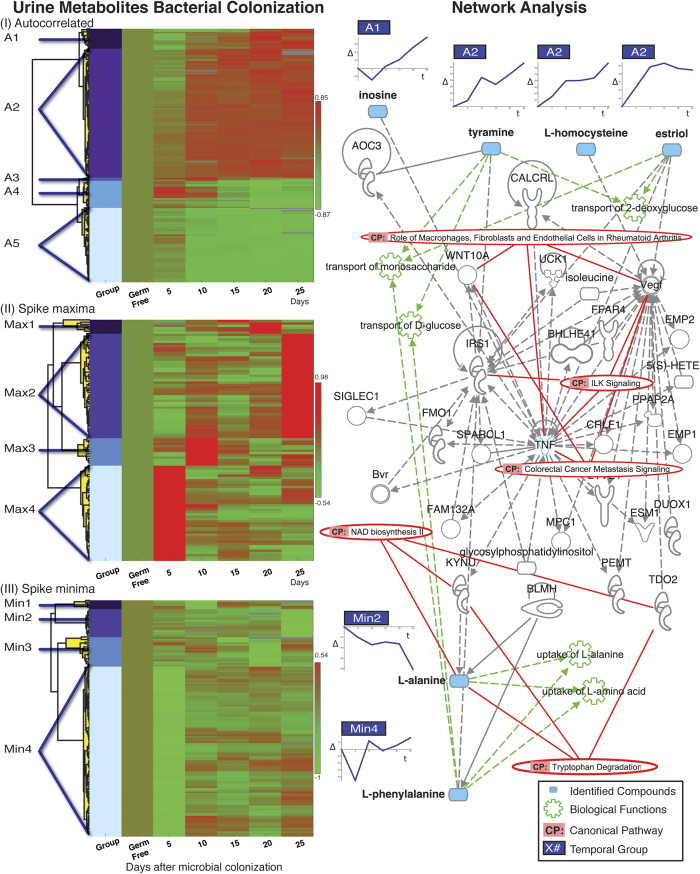
Temporal Trends and Associated Networks. On the left the hierarchical clustering per classification (Autocorrelated, Spike Maxima and Spike Minima) is shown. For each trend molecules with unique KEGG ID^72^ or identified through MS standards were used in QIAGEN’s IPA Ingenuity pathway construction (IPA^®^, QIAGEN Redwood City, www.qiagen.com/ingenuity). The network displayed on the right (network score 16, “Cell-mediated Immune Response, Inflammatory Response, Gastrointestinal Disease”) includes 5 molecules, each shown aligned horizontally with its corresponding temporal trend and identified with its group on the left. Significant functions and canonical pathway results that include more than two of the metabolites or network components respectively are also included. See also Supplementary Table S4 for detailed network composition and functional analysis and Supplementary Fig. S3.

The classification analyses of the experimental urine metabolome data assigned a total of 576 molecules to the different time trend classes (334 autocorrelated, 106 spike maxima, 136 spike minima). 45 of these molecules were considered to be high interest identifications based on their uniqueness of mass, or identity verification through the use of standards using follow-up mass spectrometry experiments (Table 1, Supplementary Table S1 for full data). Hierarchical clustering within each temporal class revealed distinct trends in the metabolite compositions, corresponding to the colonization of the GF mice (see Methods) (Fig. 3, left). The autocorrelated trends revealed two distinct groups, showing contrasting trends – one increasing constantly following colonization (A2 in Fig. 3, which included validated compounds such as tyramine, L-homocysteine, and estriol), with the other decreasing constantly (clustering group A5 in Fig. 3, including validated compounds such as 5 hydroxy-L-tryptophan and N-acetyl-L-methionine). The spike maxima and minima also displayed various trends, with the most prominent spike occurring on Day 5 (e.g. L-phenylalanine in clustering group Min 4, Fig. 3).

(3) The identified metabolites of high-interest are associated with multiple possible pathways (26 molecules were found in QIAGEN’s Ingenuity Pathways (IPA^®^, QIAGEN Redwood City, www.qiagen.com/ingenuity), Supplementary Fig. S3, including nine molecules that were verified by MS identification through comparison to standards). Examples with the lowest p-values and with more than two identified molecules involved include Amino Acid Metabolism functions, uptake of L-proline and L-alanine (p < 2.5 × 10^−5^) [both involve L-alanine and L-phenylalanine], as well as carbohydrate metabolism functions [involving tyramine and L-phenylalanine, and estriol in transport of monosaccharide (p < 4.8 × 10^−5^)], with the full IPA^®^ analysis output included in Supplementary Table S2. For the identified metabolites in the high interest list, networks were constructed algorithmically using IPA^®^ to identify connections to known gene and pathway associations. The highest scoring network (network score 16, i.e. p < 10^−16^, by IPA^®^) is shown in Fig. 3 right panel, and involves Cell-mediated Immune Response, Inflammatory Response, Gastrointestinal Disease. The network includes multiple processes such as transports of monosaccharide and D-glucose, uptake of L-alanine, L-proline, ILK signaling and associated genes, proteins and enzymes, with TNF, Vegf and IRS1 amongst the highly connected nodes (Fig. 3).

## Discussion

The microbiome is an intrinsic part of the host and should be considered in conjunction with corresponding changes in host molecular components^3^,^4^,^10^. Research is now focusing on the functional aspects of the microbiome-host interaction^54^,^55^,^56^, and reaching a stage where further progress requires systems level datasets. Such datasets will help address the global molecular interactions and resulting collective dynamic responses in metabolites, genes and proteins, and other associated omics data, and provide support for modeling^22^.

The longitudinal study presented here is a pilot implementation that has allowed us to follow the temporal changes in urine host metabolites, dynamically following colonization of GF mice. Previous experiments in germ free rats have shown that after fecal inoculation there was an increase of specific metabolites such as benzoic acid or phenylacetic acid^33^,^57^. Rather than considering individual metabolites, we have considered a collective set of all chemicals that are detected by mass spectrometry and that correspond to a temporal trend caused by the colonization. Our approach demonstrates that microbiotic changes in the gut have an immediate impact on metabolic processes, and provide readouts as collective behavior observed in urine metabolite level changes. These changes both display immediate punctuated response on the fifth day post colonization, in addition to continuous changes, both as gradual increases and decreases in the relative levels of groups of metabolites over the entire time course, suggesting longer term effects. The reported changes also indicate that gut colonization drastically alters host metabolic pathways, including carbohydrate metabolism and molecular transport, relating to host energy balance that has been previously reported in bi-species colonization studies^58^,^59^, which have been also modeled using GEMs^60^. Such modeling may be carried out for the species considered in the presented study, as well as binary and higher combinations. The overall functional association findings and possible connections to adaptive immunity are consistent with changes observed by others in longitudinal monitoring of mice and rats^11^. Other studies have verified how the presence of bacteria impact the colonic luminal metabolome^41^, as well as some endogenous metabolite levels^42^.

The observed host-microbe interaction suggests that in the absence of additional perturbation, colonization has long lasting effects on metabolic processes, which implicate several proteins/genes that participate in host immune responses. Recent mice conventionalization studies have revealed similar such immune connections, including as well the association of TNF^40^. The approach in our investigation observed the metabolic changes that resulted from the colonization by two bacteria species. This diet-induced colonization may be extended to study more bacterial species, while the use of urine as an output measure allows for generalization to human studies in a rather non-invasive fashion (compared to other body tissue sampling). Starting from different binary/paired combinations of bacterial species used for colonization, we can envision extending colonization studies systematically to triplets of species and increasingly higher numbers. If conducted in parallel with modeling approaches, this will provide the necessary data to help elucidate how metabolic processes are affected by inter-microbe dependability and how metabolism may be modulated through the introduction or removal of bacterial species.

Additionally, supplementing urine studies with monitoring of blood samples will help identify the corresponding expression changes in blood metabolomes, transcriptomes and proteomes and investigate further the connection to host immune responses. For example, other studies have reported on such omics integration that donor features can be reconstituted in conventionalized mice, and modulated through diet^61^. Such models allow an investigation of the connection of personalized diet effect mechanism, that may be studied using the few microbe approach, with extensions to pharmacomicrobiomics^20^. Furthermore, some of the validated compounds in our study such as inosine, L-Alanine and L-Phenylalanine have been reported as members of the human urine metabolome^62^, suggesting that the study of diet effected colonization in mice might have implications in similar applications to humans and modulation of the microbiotic makeup. The simplified mouse model approaches yield results that may be used to evaluate more mechanistic hypotheses^63^,^64^,^65^,^66^,^67^, including validating the algorithmic modeling of microbiome-host interactions at a systems level, for example, acting as output tests for dynamic GEM models^22^. This will also necessitate more thorough database and annotations of associations of metabolites to known genes, proteins and pathways.

The implementation of a high-throughput methodology presented here, from sample to temporal analysis, is generalizable both in sample scale and number of components and time-points. Addressing uneven sampling is of particular concerns in efforts in personalized medicine^7^,^8^,^68^, where we expect metabolomics to play a crucial role in multimodal omics integration. In mass spectrometry missing data points, which may effectively create uneven sampling are an inherent method limitation. We are able to extract patterns from noisy data (Fig. 2), allowing also for recovery of signals with missing time-points. The computational approach scales even better in longer time series (Fig. 2-c), allowing for more uneven data sampling (and more missing data points), and exploration of lags greater than one, thus extending to periodic phenomena. The generalized implementation of this framework will be included in the multi-omics analysis package *MathI-Omica* (Yusufaly and Mias, in preparation). Finally, we believe that longitudinal studies may study the different effects of microbiotic changes or the host state, and provide resource metabolite sets that correspond to each change (cf. gene set analysis generalized to small molecules).

## Methods

### Mice

All experimental protocols were in accordance with and as approved by (protocol 19727) the Administrative Panel on Laboratory Animal Care (APLAC), the Stanford Institutional Animal Care and Use Committee (IACUC). Two groups of Swiss-Webster germ free mice were used in this study. The mice were placed inside gnotobiotic isolators. One group of mice was kept GF (n = 3) and urine and feces were collected from this group. The second group of mice was colonized using oral gavage with 10^8^ cfu of *Bt* (VPI-5482) and 10^8^ cfu of *Bif. longum* (NCC2705) (n = 4). Bacteria were cultured under anaerobic conditions at 37^o^C in tryptone-yeast extract-glucose (TYG) medium and Reinforced Clostridial Medium (RCM, Becton Dickinson and Company, MD, United States). Urine and feces from this second group of bi-associated mice were collected at 5, 10, 15, 20 and 15 days after gavage. All samples were placed in a freezer at −80 °C within 30 minutes of collection, until analysis.

### Urine sample preparation and analysis^39^

Forty μl of 10 mM ammonium formate were added to 20 μl of urine. Five μl of the mix was used in the analysis. A 150 mm × 2.1 mm Kinetex 17 μm C18 column was used for chromatographic separation using a ACQUITY Ultra Performance Liquid Chromatography system (Waters). The flow rate was 0.3 ml/min with solvent A composed of water plus 0.1% formic acid and solvent B composed of acetonitrile plus 0.1% formic acid. The gradient consisted of 3% B for one min, followed by a gradient of 35% B over 15 min, hold at 35% B for 5 min, and 100% B for 2 min. The column was equilibrated at 3% B for 2 min. Mass spectrometry was performed an Exactive (Thermo Fisher) operated in positive and negative electrospray mode and controlled by Xcalibur 2.1 software. The scan range was from 70 to 800 m/z, at 50000 FWMH resolution, using nitrogen. For positive mode (ESI+) we used the following conditions: sheath gas flow rate 40 (arbitrary units), auxilary gas flow rate 8 (arbitrary units), sweep gas flow rate one (arbitrary units), spray voltage 3.5 kV, capillary temperature 275 C, capillary voltage – 60 V, tube lens voltage −100 V, skimmer voltage e −20 V. For negative electrospray mode (ESI-): sheath gas flow rate 30 (arbitrary units), auxiliary gas flow rate 4 (arbitrary units), sweep gas flow rate one (arbitrary units), spray voltage 3.5 kV, capillary temperature 275 C, capillary voltage −60 V, tube lens voltage −100 V, skimmer voltage −21 V.

### Metabolomics data processing and time analysis framework

Raw mass spectrometry spectral data were collected for each biological replicate at each time point both in positive and negative MS modes. Each dataset was analyzed as summarized in Supplementary Fig. S2: (1) preprocessed to align and assign mass intensities, (2) spectrally analyzed to obtain temporal patterns and classified into significant categories (3) assessed for assignment of mass identities and potential biological significance through pathway analysis. The primary statistical analyses were performed using Mathematica 9.0^69^ (except as indicated below). In particular, for each step:

#### Data Preprocessing

Continuous mode raw data from the individual analyses were converted to centered mode .mzXML files with msconvert (part of ProteoWizard^70^) and subjected to nornlinear data alignment by XCMS^70^,^71^. In order to identify specific biomarkers by mass, KEGG application programming interface (API) was used for identified compounds^72^, with a mass tolerance for identification set at 10 ppm. Masses uniquely identified were considered “high-priority” results.

Spectra from profiling at each time point were obtained with 3 technical replicates and aligned for mass and retention times using XCMS. The aligned spectra/mass data were filtered, and the median and median deviation were computed for replicates sets per mass identified, retaining data with a CV <0.5. For each time-point the log-2 intensities distributions were standardized to the median, scaled by the average median deviation, and only sets with mass intensities at least 2/3 time-points and including the germ-free reference were retained. For statistical computations a non-parametric bootstrap distribution with replacement was computed for 100,000 samples. For each of the constructed and experimental mass data the difference in normalized intensities was computed compared to the germ free datapoint, namely the difference, σ_Δ_ = σ_t_-σ_germ-free_, where σ_t,_ is the median deviation of each mass normalized intensity at time-point (t) using its own distribution median, and σ_germ-free_ is the median deviation the corresponding normalized mass intensity at the germ-free datapoint (germ-free mice samples), from its distribution median. The resulting time-series differences set for each mass was constructed into a normalized vector (normalized using a Euclidean distance metric).

#### Classification of Temporal Response

The normalized time series were classified based on the temporal trends observed during the time-course. To allow for missing data points (inherent in MS analyses) which essentially create uneven sampling in time, we adopted a spectral analysis for computing autocorrelations and subsequent visualization. A periodogram of the data in frequency (Fourier) space was obtained, by oversampling and using a Lomb-Scargle transformation/linear least squares harmonic function fit^45^,^46^,^47^, adapted from astronomy and utilized in biological research, which can aid in accounting for unevenly sampled data sets (e.g. due to missing datapoints)^7^,^48^,^49^,^50^,^51^,^53^,^73^,^74^,^75^,^76^. Briefly, (see also references), the Lomb-Scargle transformation computes the periodogram 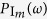 for a time series of intensities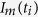 for mass, m, at the ith time point 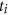



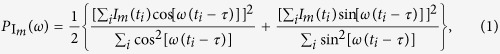


where, τ is a parameter computed implicitly,


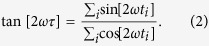


The periodogram is used together with an inverse Fourier transform to allow for even resampling and obtain the autocorrelations^46^,^47^,^48^,^53^. For each mass intensity evenly-(re)sampled time series the autocorrelation at lag one was computed, where the generalized expression


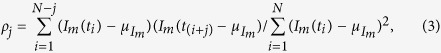


is evaluated lag j = 1. A classification scheme of autocorrelated signals and spike signals was implemented^7^, with signals having statistically significant autocorrelation classified as *autocorrelated* class (p < 0.05 cutoff, one-tailed, based on autocorrelations distribution of the bootstrap distributions simulated per data-set). The autocorrelated signals were then removed from the dataset, and signals in the remaining set showing spiky behavior of aberrantly high or low high or low normalized intensities at any time-point as compared to a random simulation were classified as *spike maxima* or *minima* (p < 0.05, one-tailed by comparison to analysis of randomly simulated distribution of normalized time signals of corresponding length N for each time-series in each class).

#### Hierarchical Clustering and Annotation

The data in each class were hierarchically clustered in *Mathematica 9.0*^69^, with (correlation distance measure with average linkage). Groups within each class were determined from the dendrograms, through changes in fusion coefficients. Within each group, the masses were annotated using the KEGG^72^ API, with a mass tolerance window of 10ppm. Additional masses of interest were compared against mass spectrometry standards for identification. Masses with unique KEGG IDs or identified through the use of standards were classified as higher priority data, and were used for pathway and network analysis through QIAGEN’s Ingenuity^®^ Pathway Analysis (IPA^®^, QIAGEN Redwood City, www.qiagen.com/ingenuity), (see Table 1). In particular, each KEGG compound identifier was mapped to its corresponding object in the IPA^®^ Knowledge Base (26 of 45 compounds). These Network Eligible molecules were used for Functional Analysis, to identify biological functions in the Ingenuity Knowledge Base. Right-tailed Fisher’s exact test was used to calculate a p-value determining the probability that each biological function assigned to the data set was due to chance alone. Additionally, canonical pathways from the IPA library that were most significant were ascertained based on p-value (Fisher’s exact test) and ratio of molecules from the data set as compared to the total in the network. Finally, a network (35 molecules) was generated using the Network Eligible molecules as seeds with IPA^®^’s Network Generation Algorithm. The network score is based on the hypergeometric distribution, calculated as –log[right-tailed Fisher’s Exact Test], (IPA^®^, QIAGEN Redwood City, www.qiagen.com/ingenuity).

## Additional Information

**How to cite this article**: Marcobal, A. *et al.* Metabolome progression during early gut microbial colonization of gnotobiotic mice. *Sci. Rep.* **5**, 11589; doi: 10.1038/srep11589 (2015).

## Supplementary Material

Supplementary Information

Supplementary Table S1

Supplementary Table S2

Supplementary Table S3

Supplementary Table S4

## Figures and Tables

**Table 1:** Possible Metabolites (Higher Priority List).

**Compound**	**KEGG ID**	**Mass (amu)**	**Class Group**
11-Deoxytetrodotoxin	C20026	302.11	A1
Kasugamycin	C17968	378.16	A1
*Inosine	C00294	267.07	A1
N-Methyl-(R,S)-tetrahydrobenzylisoquinoline	C05314	238.15	A2
Amantadine hydrochloride	C07939	186.11	A2
Glycinoeclepin A	C08765	433.25	A2
Dikegulac	C18825	275.11	A2
Longifolonine	C09569	296.10	A2
Silafluofen	C18412	407.19	A2
Mitoxantrone	C11195	445.20	A2
Naltrindole	C18128	415.19	A2
4,4-Difluoro-17beta-hydroxyandrost-5-en-3-one propionate	C15112	381.22	A2
Lycomarasmine B	C08496	276.09	A2
SR95531	C13796	288.13	A2
9alpha-Fluoro-16alpha-hydroxyhydrocortisone	C14638	397.19	A2
*L-Homocysteine	C00155	136.04	A2
*Tyramine	C00483	138.09	A2
*Estriol	C05141	287.17	A2
(2-Butylbenzofuran-3-yl)(4-hydroxyphenyl)ketone	C15049	295.13	A4
Agaritine	C01550	266.12	A4
GA	C11484	298.15	A4
Metconazole	C18476	320.15	A4
1-Carbazol-9-yl-3-(3,5-dimethylpyrazol-1-yl)-propan-2-ol	C11560	320.17	A4
L-alpha-Acetyl-N,N-dinormethadol; dinor-LAAM	C16662	326.20	A5
Hypoglycin B	C08280	271.12	A5
Dihydrozeatin riboside	C16447	352.17	A5
*5-Hydroxy-L-tryptophan	C00643	221.09	A5
*N-Acetylmethionine	C02712	192.07	A5
SN-38 carboxylate form	C11366	410.14	Max2
Fenpiclonil	C14268	234.99	Max4
Difenpiramide	C17720	289.13	Max4
Anthopleurine	C16994	176.10	Min1
N-Acetyl-beta-D-glucosaminylamine	C01239	219.11	Min2
Phenylacetylglycine dimethylamide; Ralgin	C12958	221.12	Min2
*L-Alanine	C00041	88.04	Min2
Clomipramine	C06918	315.15	Min3
3beta-Hydroxy-16-phosphonopregn-5-en-20-one monoethyl ester	C15173	425.24	Min3
Propachlor	C18759	210.08	Min4
Amantadine hydrochloride	C07939	186.11	Min4
dl-Methylephedrine hydrochloride	C13639	214.11	Min4
Streptidine	C00837	263.14	Min4
Penthiopyrad	C18482	358.13	Min4
Benalfocin; SK&F 86466	C10970	194.08	Min4
*L-Phenylalanine	C00079	166.07	Min4
*Urocanate; Urocanic acid	C00785	139.05	Min4

High confidence metabolites that showed significant temporal trends (Fig. 2). Mass features were annotated using the KEGG^72^ API, with mass tolerance 10ppm. Additional masses of interest were compared against mass spectrometry standards for identification. Masses with unique KEGG IDs or identified through the use of standards were classified as high priority and were used for pathway and network analysis through QIAGEN’s Ingenuity^®^ Pathway Analysis (IPA^®^, QIAGEN Redwood City, www.qiagen.com/ingenuity). See also Supplementrary Fig. S2 for analysis framework details and Supplementary Table S3 for full classification data.

^*^were identified by use of standards using LC-MS.
