# Supplementary material for: Metabolome progression during early gut microbial colonization of gnotobiotic mice
Source: Sci Rep. 2015 Jun 29;5:11589. doi: 10.1038/srep11589 (PMC4484351; doi:10.1038/srep11589)
Supplement: Supplementary Information [file srep11589-s1.pdf]

SUPPLEMENTARY INFORMATION

Metabolome progression during early gut microbial colonization of gnotobiotic mice

Angela Marcobal[1], Tahir Yusufaly[2], Steven Higginbottom[1], Michael Snyder[3], Justin L. Sonnenburg[1, 4], and George I. Mias[2, 4].

[1] Department of Microbiology and Immunology, Stanford University School of Medicine, Stanford, California, USA,

[2] Department of Genetics, Stanford University School of Medicine, Stanford, CA, USA

[3] Department of Biochemistry and Molecular Biology, Michigan State University, East Lansing, Michigan, USA.

[4] J.L.S. and G.I.M. are both senior authors.

Correspondence should be addressed to G.I.M. (gmias@msu.edu) or J.L.S. (jsonnenburg@stanford.edu).

SUPPLEMENTARY INFORMATION 1

Supplementary Figures 3

Supplementary Figure S1. Bacterial Composition. 3

Supplementary Figure S2. Analysis Framework 4

Supplementary Figure S3. Metabolite Annotations. 6

Supplementary Table/Dataset Information 8

Supplementary Bibliography 9

Supplementary Figures

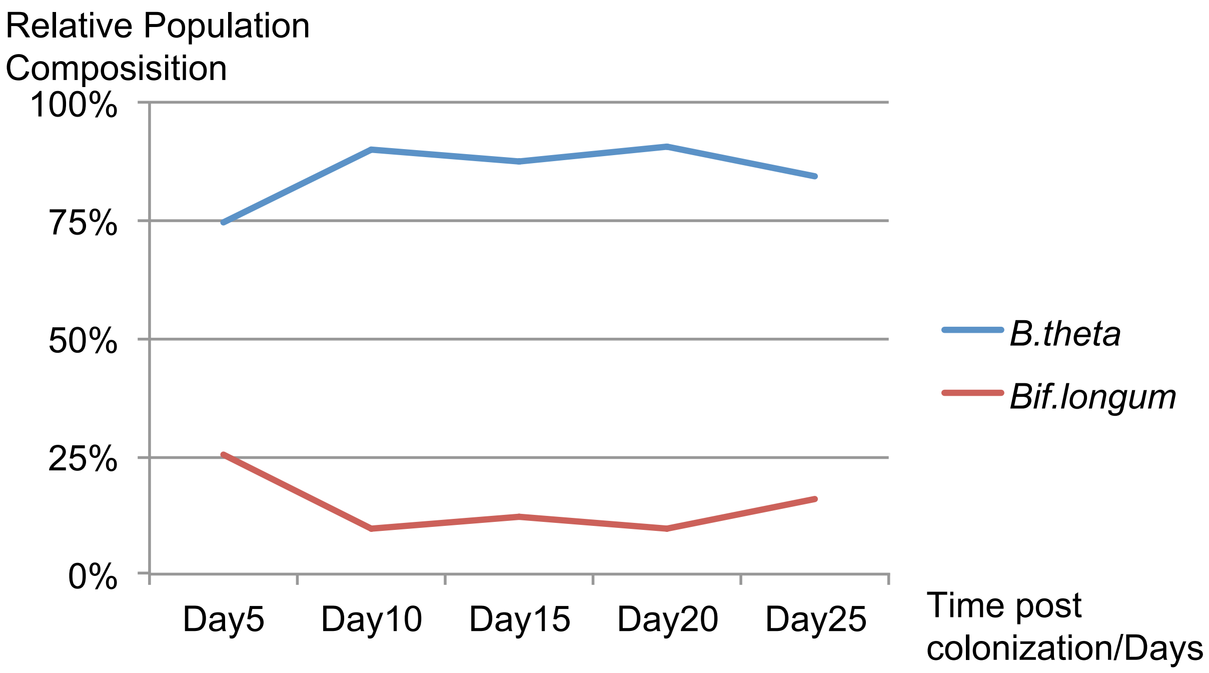

Supplementary Figure S1. Bacterial Composition.

The bacterial composition of the gut microbiota was assessed using fecal samples.

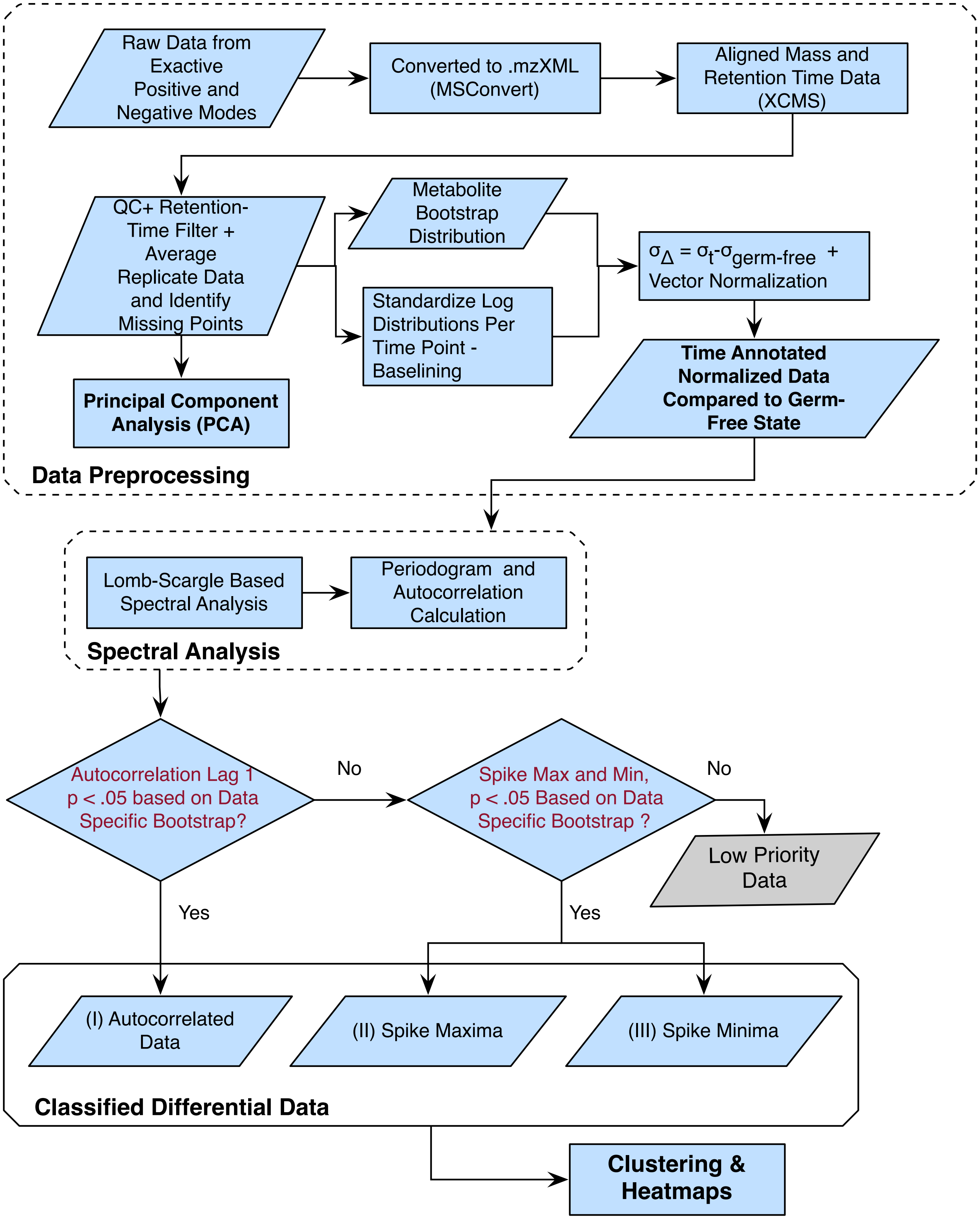

Supplementary Figure S2. Analysis Framework

The raw data files are processed in a three-step process. Initially the raw data is preprocessed

for both negative and positive MS acquisition modes. The data is converted to mzXML[1] using MSConvert[2] and the spectral and retention time information is aligned with XCMS[3]. After quality control, including replicate processing the log intensity distributions are standardized and also a bootstrap distribution is constructed. The differences of individual mass intensities are calculated with respect to the germ-free time point (Day 0) and a normalized time series set constructed for each mass label. In the second step the periodogram and autocorrelations of the data are computed in frequency space using Fourier analyses. In step three the data is classified into autocorrelated and two spike classes if it a series displays autocorrelations or aberrant increases (spike maxima) or decreases (spike minima) at specific time-points compared to what one would expect from a random distribution. The data within each class is then clustered using hierarchical algorithms and annotated (see also Fig. 2-3 and Supplementary Fig. S3).

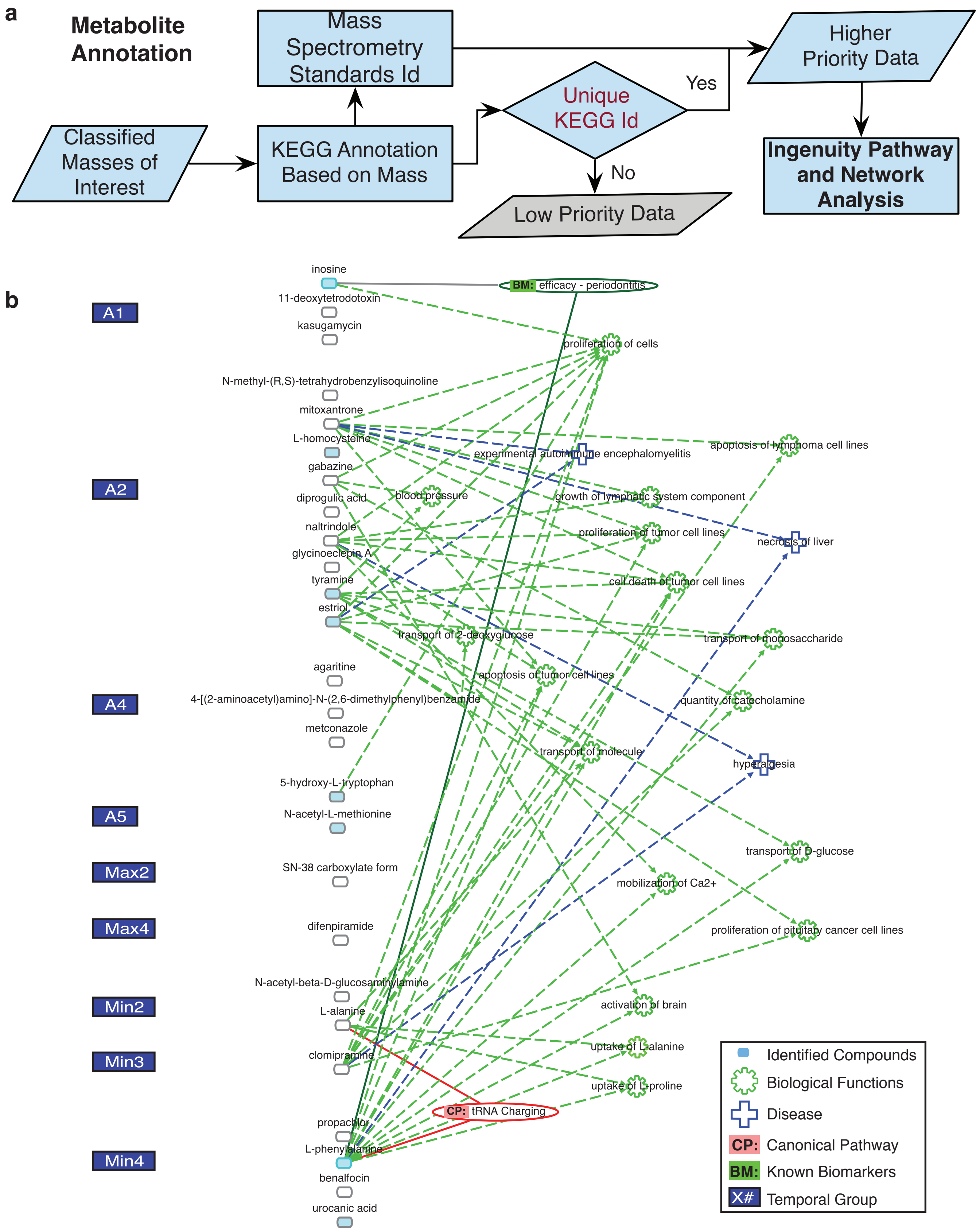

Supplementary Figure S3. Metabolite Annotations.

(a) The masses for each group per classification were annotated using the KEGG[4] database,

or verified using standards and a new round of mass spectrometry. Higher priority data included masses with unique KEGG[4] Ids or MS-verified, and were used in QIAGEN's Ingenuity® Pathways and Network Analysis Analysis (IPA®, QIAGEN Redwood City, www.qiagen.com/ingenuity). **(b)** Twenty six KEGG[4] annotations were in the Ingenuity Knowledge database and were used for function, canonical pathway and biomarker and enrichment analysis (a minimum of two connections was required for the displayed data). Each molecule displayed different molecular trends, with the Temporal Groups annotated as identified in Fig. 3 and Table 1**.**

Supplementary Table/Dataset Information

The following are included as Excel spreadsheets:

Supplementary Table/Dataset S1. Negative and Positive Mode MS Data aligned by XCMS.

Supplementary Table/Dataset S2. Full QIAGEN's IPA® Analysis.

Supplementary Table/Dataset S3. Metabolite Classification Annotation.

Supplementary Table/Dataset S4. QIAGEN's IPA® Ingenuity Analysis Networks.

Supplementary Bibliography

1 Pedrioli, P. G. *et al.* A common open representation of mass spectrometry data and its application to proteomics research. *Nat. Biotechnol.* **22**, 1459-1466, doi: 10.1038/nbt1031 (2004).

2 Kessner, D., Chambers, M., Burke, R., Agus, D. & Mallick, P. ProteoWizard: open source software for rapid proteomics tools development. *Bioinformatics* **24**, 2534-2536, doi:10.1093/bioinformatics/btn323 (2008).

3 Smith, C. A., Want, E. J., O'Maille, G., Abagyan, R. & Siuzdak, G. XCMS: processing mass spectrometry data for metabolite profiling using nonlinear peak alignment, matching, and identification. *Anal. Chem.* **78**, 779-787, doi:10.1021/ac051437y (2006).

4 Kanehisa, M. & Goto, S. KEGG: kyoto encyclopedia of genes and genomes. *Nucleic Acids Res.* **28**, 27-30, doi:10.1093/nar/28.1.27 (2000).
